# Supplementary material for: Comparative efficacy of different types of acupuncture as adjuvant therapy on carotid atherosclerosis: a protocol for systematic review and network meta-analysis
Source: BMJ Open. 2022 Apr 5;12(4):e049778. doi: 10.1136/bmjopen-2021-049778 (PMC8987791; doi:10.1136/bmjopen-2021-049778)
Supplement: Supplementary data [file bmjopen-2021-049778supp001.pdf]

Search strategy for EMBASE

Number	Search items
#1	"Atherosclerosis"/exp
#2	Atheroscleroses: ab,ti
#3	Atherogenesis: ab,ti
#4	"Carotid atherosclerosis": ab,ti
#5	#1 or #2 or #3 or #4
#6	"Acupuncture"/exp
#7	"Acupuncture Therapy": ab,ti
#8	"Acupuncture, Ear": ab,ti
#9	"Acupuncture Points": ab,ti
#10	Electroacupuncture: ab,ti
#11	"Auricular point": ab,ti
#12	#6 or #7 or #8 or #9 or #10 or #11
#13	"Statins"/exp
#14	atorvastatin: ab,ti
#15	aspirin: ab,ti
#16	#13 or #14 or #15
#17	"randomized controlled trial"/exp
#18	"randomized controlled trial": ab,ti
#19	randomized: ab,ti
#20	randomly: ab,ti
#21	"controlled clinical trial": ab,ti
#22	"clinical trial": ab,ti
#23	trial: ab,ti
#24	#17 or #18 or #19 or #20 or #21 or # 22# 23
#25	#5 and #12 and #16 and # 24

Search strategy for Medline database

Atherosclerosis		Acupuncture		Statins		'Randomized Controlled Trial'
OR		OR		OR		OR
Atheroscleroses		'Acupuncture Therapy'		Atorvastatin		randomized
OR		OR		OR		OR
Atherogenesis	AND	'Acupuncture, Ear'	AND	Aspirin	AND	randomly
OR		OR				OR
'Carotid atherosclerosis'		'Acupuncture Points'				'Controlled Clinical Trial'
		OR				OR
		'Electroacupuncture'				'clinical trial'
		OR				OR
		'Auricular point'				trial

Search strategy for Cochrane Library	
Number	Search items
#1	MeSH descriptor: [Atherosclerosis] explode all trees
#2	Atheroscleroses: ti,ab,kw
#3	Atherogenesis: ti,ab,kw
#4	"Carotid atherosclerosis": ti,ab,kw
#5	#1 or #2 or #3 or #4
#6	MeSH descriptor: [Acupuncture] explode all trees
#7	"Acupuncture Therapy": ti,ab,kw
#8	"Acupuncture, Ear": ti,ab,kw
#9	"Acupuncture Points": ti,ab,kw
#10	Electroacupuncture: ti,ab,kw
#11	"Auricular point": ti,ab,kw
#12	#6 or #7 or #8 or #9 or #10 or #11
#13	MeSH descriptor: [Statins] explode all trees
#14	atorvastatin: ti,ab,kw
#15	aspirin: ti,ab,kw
#16	#13 or #14 or #15
#17	MeSH descriptor: [Randomized Controlled Trial] explode all trees
#18	"Randomized Controlled Trial": ti,ab,kw
#19	randomized: ti,ab,kw
#20	randomly: ti,ab,kw
#21	"controlled clinical trial": ti,ab,kw
#22	"clinical trial": ti,ab,kw
#23	trial: ti,ab,kw
#24	#17 or #18 or #19 or #20 or #21 or # 22# 23
#25	#5 and #12 and #16 and # 24

Search strategy for CNKI
（主题或摘要：动脉粥样硬化+动脉硬化+斑块+颈动脉粥样硬化+颈动脉硬化+颈动脉）AND（主题或摘要：针刺+针灸+电针+温针灸+耳针+耳穴+穴位）AND（主题或摘要：他汀类药物+阿司匹林）AND（主题或摘要：随机对照研究+随机+对照研究+对照+试验+临床研究）

Search strategy for WF
（主题：动脉粥样硬化+动脉硬化+斑块+颈动脉粥样硬化+颈动脉硬化+颈动脉）AND（主题：针刺+针灸+电针+温针灸+耳针+耳穴+穴位）AND（主题：他汀类药物+阿司匹林）AND（主题：随机对照研究+随机+对照研究+对照+试验+临床研究）

Search strategy for CQVIP
（题名或关键词：动脉粥样硬化+动脉硬化+斑块+颈动脉粥样硬化+颈动脉硬化+颈动脉）AND（题名或关键词：针刺+针灸+电针+温针灸+耳针+耳穴+穴位）AND（题名或关键词：他汀类药物+阿司匹林）AND（题名或关键词：随机对照研究+随机+对照研究+对照+试验+临床研究）
