# Supplementary material for: Comparative efficacy of different types of acupuncture as adjuvant therapy on carotid atherosclerosis: a protocol for systematic review and network meta-analysis
Source: BMJ Open. 2022 Apr 5;12(4):e049778. doi: 10.1136/bmjopen-2021-049778 (PMC8987791; doi:10.1136/bmjopen-2021-049778)
Supplement: Author's manuscript [file bmjopen-2021-049778.draft_revisions.pdf]

BMJ Open is committed to open peer review. As part of this commitment we make the peer review history of every article we publish publicly available.

When an article is published we post the peer reviewers' comments and the authors' responses online. We also post the versions of the paper that were used during peer review. These are the versions that the peer review comments apply to.

The versions of the paper that follow are the versions that were submitted during the peer review process. They are not the versions of record or the final published versions. They should not be cited or distributed as the published version of this manuscript.

BMJ Open is an open access journal and the full, final, typeset and author-corrected version of record of the manuscript is available on our site with no access controls, subscription charges or pay-per-view fees (http://bmjopen.bmj.com).

If you have any questions on BMJ Open's open peer review process please email info.bmjopen@bmj.com

BMJ Open

The comparative efficacy of different acupuncture as adjuvant therapy on carotid atherosclerosis: A protocol for systematic review and network meta-analysis

Journal:	*BMJ Open*
Manuscript ID	bmjopen-2021-049778
Article Type:	Protocol
Date Submitted by the Author:	04-Feb-2021
Complete List of Authors:	Wu, xianming; Hunan University of Chinese Medicine<br>Mo, Qian; Guizhou University of Traditional Chinese Medicine<br>Yang, Zhihong; Guizhou University of Traditional Chinese Medicine<br>Huang, Xiaolou; Guizhou University of Traditional Chinese Medicine<br>Liu, Jiao; Guizhou University of Traditional Chinese Medicine<br>Xu, Shuangmei; Guizhou University of Traditional Chinese Medicine<br>Zhang, Ning; Guizhou University of Traditional Chinese Medicine<br>Yang, Xiaofang; Hunan University of Chinese Medicine; Guizhou University Of Traditional Chinese Medicine
Keywords:	COMPLEMENTARY MEDICINE, CARDIOLOGY, VASCULAR MEDICINE

SCHOLARONE™
Manuscripts

The comparative efficacy of different acupuncture as adjuvant therapy on carotid atherosclerosis: A protocol for systematic review and network meta-analysis

Xianming Wu,[1] Qian Mo,[2] Zhihong Yang,[2] Xiaolou Huang,[2] Jiao Liu,[2] Shuangmei Xu,[2] Ning Zhang,[2*] Xiaofang Yang,[1, 2 *]

[1] Hunan University of Chinese Medicine, Changsha, China

[2] Guizhou University of Traditional Chinese Medicine, Guiyang, China

XW and QM contributed equally to this paper.

Correspondence to Ning Zhang and Xiaofang Yang; 178144416@qq.com, Yangxiaofang210@163.com; Dongqing South Road, Huaxi University Town, Gui'an New District, Guizhou Province, China.

For peer review only

Abstract

Introduction Carotid atherosclerosis (CAS) is a disease of the aorta caused by lipid metabolism disorders and local inflammation. Acupuncture combined with traditional western medicine (such as aspirin or atorvastatin) for the treatment of CAS has been widely applied in clinical practice, but there is still a lack of supporting evidence for the efficacy and safety of CAS. Therefore, this systematic review and network meta-analysis (NMA) will summarize the effects of different types of acupuncture treatments on CAS, and a ranking of the therapeutic classes will also be presented, aiming to provide evidence-based medicine for its extensive clinical application.

Methods and analysis Systematic and NMA searches will be conducted in eight electronic databases, PubMed, EMBASE, Medline, Cochrane Library, Chinese National Knowledge Infrastructure (CNKI), Wan fang Database, and Chongqing VIP databases (CQVIP). The search time is from their inception to December 2020, regardless of language and publication type. Randomized controlled trials (RCTs) that include patients with CAS receiving acupuncture therapy compared with a control group will be considered eligible. The primary outcomes include the carotid intima-media thickness (IMT), the secondary outcomes include the carotid plaque Crouse score and plaque area, blood lipid, the incidence of cardiovascular events, safety, and adverse events. The selection of studies, data extraction, quality

assessment, and risk of bias assessment will be conducted by two independent reviewers. The NMA will be analyzed with Stata V.15.0, RevMan V.5.3 software, and WinBUGS 1.4.3.

Ethics and dissemination Ethical approval will not be required for this study as it will be based on de-identified, aggregate published data. We will publish the findings in a peer-reviewed journal.

PROSPERO registration number CRD42020207260.

Strengths and limitations of this study

1. This study will be the first comprehensive analysis of the efficacy of acupuncture as an adjuvant therapy in the treatment of atherosclerosis. And a ranking of the therapeutic classes will also be presented.

2. The data report will follow the Preferred Reporting Items of the System review and the Meta-analysis guidelines.

3. This study only includes English and Chinese trials, which may lead to the potential risk of ignoring some studies.

4. The heterogeneity of different studies may affect the final results of this study.

INTRODUCTION

Atherosclerosis (AS) is a series of pathological changes in the vascular intima through lipid infiltration, platelet activation, thrombosis, intimal injury, inflammatory response, oxidative stress, and activation of vascular smooth muscle cells (VSMC). It is manifested by cell metabolism disorder, product accumulation, which causes the vessel wall to harden and thicken, and lose its elasticity, the inner diameter of the lumen becomes smaller, blood flow is blocked, and finally, the diseased blood vessel blood supply organs and even life are endangered.[1-3] The World Health Organization released the National Survey of Non-communicable Diseases in 2018, showing that my country's non-communicable disease mortality rate is estimated to account for 89% of all deaths, of which cardiovascular disease accounts for 43% of the proportion of non-communicable diseases. Globally, the number of people dying from cardiovascular disease is still increasing. In response to this phenomenon, the United Nations has set a goal and plans to adopt various measures to make 30 - 70 years old people die prematurely due to cardiovascular disease by 2025. 25% reduction in mortality.[4] Among them, carotid artery atherosclerosis (CAS) has common risk factors and

For peer review only - http://bmjopen.bmj.com/site/about/guidelines.xhtml

pathological basis with atherosclerosis of the coronary arteries of the heart, cerebral blood vessels, and renal arteries.[5-7] A study has found that 93% of cerebral infarctions are accompanied by CAS, and 18%-25% of ischemic strokes can be attributed to thromboembolism caused by CAS[8].

The current conventional western medical treatments for CAS are mainly hypolipidemic, antiplatelet, etc. (such as aspirin or atorvastatin).[9] Statins can treat AS by regulating blood lipids, improving vascular endothelial function, inhibiting thrombosis, anti-inflammatory, anti-oxidation, and stabilizing plaque.[10] Acupuncture is part of the characteristic treatment of traditional Chinese medicine. Its main feature is to stimulate the meridians and acupoints and regulate the qi and blood of the viscera to achieve the purpose of disease prevention and treatment.[11] At present, acupuncture is widely used as an adjuvant treatment of AS as a characteristic treatment method of Chinese medicine.[12,13]

Although there have been RCTs to study the efficacy of acupuncture intervention on AS, due to sample size and insufficient randomization methods in clinical trial studies, and the different objectives and outcome evaluation indicators used, the results of the research are not consistent. What type of acupuncture method is better to prevent, stabilize and reverse CAS is still inconclusive. Therefore, this study uses a network meta-analysis to summarize the effects of different types of acupuncture treatments on CAS and aims to provide evidence-based medicine for the extensive clinical use of acupuncture as an adjuvant therapy to treat AS.

Objectives

The objectives of our study are:

1. To evaluate the efficacy and safety of acupuncture as adjuvant therapy in the treatment of AS.

2. To present the ranking of therapeutic classes.

METHODS

Study registration

This NMA will be performed according to the guidelines of preferred reporting items for systematic reviews and meta-analyses (PRISMA-NMA) statement.[14] The protocol has been registered on the PROSPERO website (https://www.crd.york.ac.uk/prospero/) and the PROSPERO registration number is CRD42020207260.

For peer review only - http://bmjopen.bmj.com/site/about/guidelines.xhtml

Data sources and search strategy

Seven electronic databases including PubMed, EMBASE, Medline, Cochrane Library, Chinese National Knowledge Infrastructure (CNKI), Wan fang Database, and Chongqing VIP databases (CQVIP) will be searched from inception to December 2020. The search strategy will be adjusted according to the characteristics of the database. The detailed search strategy of PubMed will be shown in Table 1.

Table 1 Search strategy used in PubMed database.

Number	Search items
#1	Atherosclerosis[Mesh]
#2	Atheroscleroses [Title/Abstract]
#3	Atherogenesis [Title/Abstract]
#4	Carotid atherosclerosis [Title/Abstract]
#5	#1 or #2 or #3 or #4
#6	Acupuncture[Mesh]
#7	Acupuncture Therapy [Title/Abstract]
#8	Moxibustion [Title/Abstract]
#9	Acupuncture, Ear [Title/Abstract]
#10	Acupuncture Points [Title/Abstract]
#11	Electroacupuncture [Title/Abstract]
#12	Auricular point [Title/Abstract]
#13	#6 or #7 or #8 or #9 or #10 or #11 or #12
#14	Statins [Title/Abstract]
#15	atorvastatin [Title/Abstract]
#16	aspirin [Title/Abstract]
#17	#14 or #15 or #16
#18	randomized controlled trial[Publication Type]
#19	controlled clinical trial[Title/Abstract]
#20	randomized[Title/Abstract]
#21	randomly[Title/Abstract]
#22	#15 or #16 or #17 or #18
#23	#5 and #13 and #17 and #22

Inclusion and exclusion criteria

Types of studies

All RCTs will be included and the full text is available, the language is limited to Chinese and English, regardless of publication type. Retrospective studies, case reports, reviews, or studies on the mechanism of action will be excluded.

Participants

For peer review only

For peer review only - http://bmjopen.bmj.com/site/about/guidelines.xhtml

Studies that enrolled patients were diagnosed with CAS, without serious liver and kidney dysfunction and tumor patients. while gender, age, ethnicity, and nationality will not be limited. The baseline data of the observation group and the control group are comparable. We will follow the relevant standards of the guidelines for atherosclerotic vascular ultrasound examination[9].

Types of interventions

Both the observation group and the control group were treated with conventional western medicine, including aspirin or atorvastatin, etc. The intervention of the observation group was to add acupuncture to the conventional Western medicine treatment. Acupuncture therapies included acupuncture, electro-acupuncture, auricular acupuncture, moxibustion, warm moxibustion, etc., without restrictions of acupuncture and moxibustion operation methods, acupoint selection, duration of treatment, and follow-up period.

Types of comparison

The control group will include treatments of placebo or the same western medicine treatment in the observation group, such as aspirin or atorvastatin.

Outcomes

The primary outcome indicator will be the changes of IMT before and after treatment.

The secondary outcomes include the changes of carotid plaque Crouse score and plaque area. Safety outcomes include the type and frequency of occurrence of adverse reactions, etc. such as subcutaneous hematoma, fatigue, palpitations, etc.

Selection of studies.

According to the inclusion and exclusion criteria, two independent reviewers (XMW and QM) will remove duplicates and unqualified articles after reading the titles and abstract. For articles that meet the inclusion criteria, the full texts will be evaluated for eligibility. If there is any disagreement between the two reviewers, the third reviewer (XFY) will make a final decision after discussion. If the data or information are incomplete, the authors will be contacted. The PRISMA flow diagram (Figure 1) will be used to show the research screening process.

Data extraction and management

For peer review only - http://bmjopen.bmj.com/site/about/guidelines.xhtml

All reviewers discussed and developed data extraction forms based on the Cochrane Handbook, and two independent reviewers (XMW and QM) will extract data from the included studies. The extracted data includes general information (author, publication year, journal, etc.), participant, sample size, randomness, blinding, allocation hiding, intervention methods, results, follow-up time, etc. If the data is incomplete, the corresponding author will be contacted. Multi-arm trials will be assigned into two-arm to ensure that the results can be combined.[15]

Risk of bias assessment

Two reviewers (XMW and QM) will use the "Risk of bias" tool from the Cochrane Handbook (V.5.1.0) to assess the quality of the included studies.[14,16] The contents of the evaluation include random sequence generation, allocation concealment, and implementation of blinding, incomplete outcome data, selective reports, and other issues. Any inconsistency will be decided through discussion with the third reviewer (XFY). The risk of bias will be classified as "high risk of bias", "low risk of bias" or "unclear risk of bias".

STATISTICAL ANALYSIS

Standard pairwise meta-analysis

Standard pairwise meta-analysis was performed using Stata V.15.0 software. The odds ratio (OR) was used as the effect size for count data, the mean difference was used for measurement data, and 95% confidence interval (CI) was used for interval estimation. According to the "Cochrane Handbook", the Mantel-Haenszel χ^2 test and Higgins I^2 test will analyze the heterogeneity. P-value <.10 or I^2 >50% indicates that the heterogeneity is statistically significant, and the source of the heterogeneity is analyzed. And according to the possible heterogeneity factors, conduct subgroup analysis, and then sensitivity analysis was carried out to ascertain the reliability of the data, find abnormal studies that lead to significant heterogeneity, and analyze the reasons. Heterogeneity also depends on the size of the impact, the direction of the results, and the strength of the evidence.

Network meta-analysis and network geometry

WinBUGS 1.4.3 will be used for network statistical analysis,[17] and the calculation will be performed by the Markov chain Monte Carlo method. Calculate the surface under cumulative ranking area (SUCRA) to predict the curative effect of each treatment measure and rank it.

For peer review only - http://bmjopen.bmj.com/site/about/guidelines.xhtml

The result is expressed as a percentage, the larger the value, the better the curative effect of the intervention.[18] Stata 15.0 draws a network geometry, the thickness of the arm represents the number of studies, the area of the circle represents the size of the sample.[19,20] The comparisons adjusted funnel plot was used to compare sample effects between studies and evaluate the publication bias of the included studies.

Assessment of inconsistency

We will use loop-specific methods to detect loops of evidence that may have important inconsistencies.[21] A node split model is used to compare the consistency of direct comparison and indirect comparison,[22] P>0.05 means that the consistency between direct and indirect comparison is better, consistency model is used for analysis. Otherwise, the inconsistency model is used. For those that have not generated node split, the consistency model is used for analysis.

Subgroup and sensitivity analyses

To assess the impact of covariates in heterogeneity, such as gender, disease severity, we will explore subgroup analysis.

Subgroup analysis still has obvious heterogeneity, and sensitivity analysis will be performed. After excluding low-quality studies in order, the meta-analysis will be repeated and the results of the two meta-analyses will be compared to identify the impact of each study on the overall results.

Assessment of publication biases

If more than 10 trials are included, a Deek's funnel plot to evaluate publication bias. If the angle between the regression line and the X-axis is closer to 90°, it means that the less possibility of publication bias.[23]

Ethics and dissemination

Since the study will be based on de-identified, aggregate published data, ethical approval is not required. The final report of this review will be disseminated through peer-reviewed publications or conference reports.

Patient and public involvement

No patient or public will be involved in the study directly.

DISCUSSION

For peer review only - http://bmjopen.bmj.com/site/about/guidelines.xhtml

CAS is a common multiple diseases that endangers human health. The common pathological basis of cardiovascular and cerebrovascular diseases has become the main cause of death in the world. Clinically, the treatment of AS is roughly divided into drugs and non-drug treatments. Among them, drug treatments include statins, which have anti-platelet, anti-inflammatory, lipid-regulating, and inhibit angiogenesis effects in plaques.[24,25] Although great progress has been made in the treatment of AS, the complex and diverse causes and mechanisms of AS (closely related to metabolic, environmental, genetic, and other factors) make the existing clinical intervention methods. The treatment of AS is still insufficient, and diseases caused by AS still bring huge health and economic burden to society.[26] In recent years, acupuncture has been widely used as an adjuvant treatment of AS. However, there is no research to rank acupuncture interventions and evaluate the optimal acupuncture methods. Therefore, this network meta-analysis will conduct a detailed summary and analysis of acupuncture as adjuvant therapy for CAS to determine the optimal acupuncture therapy.

The comparison between therapies in the network meta-analysis is based on direct comparisons of interventions within RCTs and indirect comparisons across trials based on a common comparator, such as placebo or some standard treatment. The interrelationship between multiple interventions can be analyzed, and deal with factors that may have an impact due to the number of included articles, heterogeneity, and inconsistency.[27,28]

Nevertheless, this network meta-analysis still has limitations. Some low-quality trials may affect the final results of the NMA. In addition, the heterogeneity of different studies may affect the final results of this study. Finally, this study only includes English and Chinese trials, which may lead to the potential risk of ignoring some studies.

Despite the limitations, the latest data should be systematically reviewed. It is concluded that different types of acupuncture can assist patients with CAS to better improve carotid IMT, and can provide evidence-based medicine for clinical evidence.

Contributors XW and QM conceived of the protocol and drafted the manuscript. XW and TL registered the protocol review in the PROSPERO, ZY and XH developed the search strategy, NZ revised the manuscript for methodological and intellectual content. XY contributed to and approved the final manuscript of the protocol review.

Funding This study is supported by National Natural Science Foundation of China (grant number:

For peer review only - http://bmjopen.bmj.com/site/about/guidelines.xhtml

81460703).

Competing interests None declared.

Patient and public involvement Patients and/or the public were not involved in the design, or conduct, or reporting, or dissemination plans of this research.

Patient consent for publication Not required.

Provenance and peer review Not commissioned; externally peer reviewed.

Open access This is an open access article distributed in accordance with the Creative Commons Attribution Non Commercial (CC BY- NC 4.0) license, which permits others to distribute, remix, adapt, build upon this work non- commercially, and license their derivative works on different terms, provided the original work is properly cited, appropriate credit is given, any changes made indicated, and the use is non- commercial. See: http:// creativecommons. org/ licenses/ by- nc/ 4. 0/.References

ORCID iD

Xianming Wu https://orcid.org/0000-0002-4825-0944

REFERENCES

1 Xu S, Kamato D, Little PJ, Nakagawa S, Pelisek J, Jin ZG. Targeting epigenetics and non-coding RNAs in atherosclerosis: from mechanisms to therapeutics. *Pharmacol Ther.* 2019;196:15-43.

2 Zhu Y, Xian X, Wang Z, et al. Research Progress on the Relationship between Atherosclerosis and Inflammation. *Biomolecules.* 2018;8(3):80.

3 Tall AR, Yvan-Charvet L. Cholesterol, inflammation and innate immunity. *Nature reviews Immunology.* 2015;15(2):104-116.

4 Roth GA, Forouzanfar MH, Moran AE, et al. Demographic and epidemiologic drivers of global cardiovascular mortality. *N Engl J Med.* 2015;372(14):1333-1341.

5 Zhao D, Liu J, Wang M, Zhang X, Zhou M. Epidemiology of cardiovascular disease in China: current features and implications. *Nat Rev Cardiol.* 2019;16(4):203-212.

6 Kobiyama K, Ley K. Atherosclerosis. *Circulation research.* 2018;123(10):1118-1120.

7 Libby P, Buring JE, Badimon L, et al. Atherosclerosis. *Nature reviews Disease primers.* 2019;5(1):56.

8 Ooi YC, Gonzalez NR. Management of extracranial carotid artery disease. *Cardiology clinics.* 2015;33(1):1-35.

For peer review only - http://bmjopen.bmj.com/site/about/guidelines.xhtml

9 Kinoshita M, Yokote K, Arai H, et al. Japan Atherosclerosis Society (JAS) Guidelines for Prevention of Atherosclerotic Cardiovascular Diseases 2017. *Journal of atherosclerosis and thrombosis.* 2018;25(9):846-984.

10 Liu M, Liu G, Wang M. Comparison and analysis of the therapeutic effect of different statins in the treatment of atherosclerosis. *Pakistan journal of pharmaceutical sciences.* 2018;31 (4):1737-1742.

11 Zhang YP LY, Xu Y, et al. Preliminary Analysis on Anti-inflammatory Mechanism of Acupuncture. *Journal of Clinical Acupuncture and Moxibustion* 2018;34(01):77-80.

12 Shen Y, Cheng ZD, Chen YG, et al. Electroacupuncture Inhibits Atherosclerosis through Regulating Intestinal Flora and Host Metabolites in Rabbit. *Evidence-based complementary and alternative medicine : eCAM.* 2020;2020:5790275.

13 HT L, LC M, Y Y, et al. Clinical Observation on Acupuncture Treatment of Carotid Atherosclerosis of Phlegm-Damp Constitution. *Guangdong Medical Journal.* 2018;39(13):2070-2073. (Chinese)

14 Julian PT Higgins SG. Cochrane Handbook for Systematic Reviews of Interventions Version 5.1.0 2011.

15 Zhang C, Yan JZ, Sun F, Liu Q, Zeng XT. Dif erentiation and Handling of Homogeneity in Network Meta-analysis. *Chinese Journal of Evidence-Based Medicine.* 2014;14(7):884-888.

16 Guyatt G, Busse J. Tools to assess risk of bias in randomized controlled trials. In:2015.

17 Dave V, Yadav RB, Yadav S, Sharma S, Sahu RK, Ajayi AF. A Critique of Computer Simulation Software's Used in Pharmacokinetics and Pharmacodynamics Analysis. *Current clinical pharmacology.* 2018;13(4):216-235.

18 Cope S, Jansen JP. Quantitative summaries of treatment effect estimates obtained with network meta-analysis of survival curves to inform decision-making. *BMC Med Res Methodol.* 2013;13:147.

19 van Valkenhoef G, Lu G, de Brock B, Hillege H, Ades AE, Welton NJ. Automating network meta-analysis. *Research synthesis methods.* 2012;3(4):285-299.

20 Nyaga VN, Aerts M, Arbyn M. ANOVA model for network meta-analysis of diagnostic test accuracy data. *Statistical methods in medical research.* 2018;27(6):1766-1784.

21 Bucher HC, Guyatt GH, Griffith LE, Walter SD. The results of direct and indirect treatment

For peer review only - http://bmjopen.bmj.com/site/about/guidelines.xhtml

comparisons in meta-analysis of randomized controlled trials. *Journal of clinical epidemiology.* 1997;50(6):683-691.

22 Dias S, Welton NJ, Caldwell DM, Ades AE. Checking consistency in mixed treatment comparison meta-analysis. *Stat Med.* 2010;29(7-8):932-944.

23 Deeks JJ, Macaskill P, Irwig L. The performance of tests of publication bias and other sample size effects in systematic reviews of diagnostic test accuracy was assessed. *Journal of clinical epidemiology.* 2005;58(9):882-893.

24 Kirichenko TV, Sobenin IA, Nikolic D, Rizzo M, Orekhov AN. Anti-cytokine therapy for prevention of atherosclerosis. *Phytomedicine : international journal of phytotherapy and phytopharmacology.* 2016;23(11):1198-1210.

25 Khosravi M, Poursaleh A, Ghasempour G, Farhad S, Najafi M. The effects of oxidative stress on the development of atherosclerosis. *Biological chemistry.* 2019;400(6):711-732.

26 Bubnova MG, Kukharchuk VV. [Recommendations of the European Society of Cardiology and the European Atherosclerosis Society on Cardiovascular Disease Prevention and Management of Dyslipidemias. for the Diagnosis of Atherosclerosis and Dyslipidemia Treatment (2016): Basic S.G.]. *Kardiologiia.* 2017;57(3):85-89.

27 Li T, Puhan MA, Vedula SS, Singh S, Dickersin K. Network meta-analysis-highly attractive but more methodological research is needed. *BMC medicine.* 2011;9:79.

28 Mills EJ, Thorlund K, Ioannidis JP. Demystifying trial networks and network meta-analysis. *BMJ (Clinical research ed).* 2013;346:f2914.

For peer review only - http://bmjopen.bmj.com/site/about/guidelines.xhtml

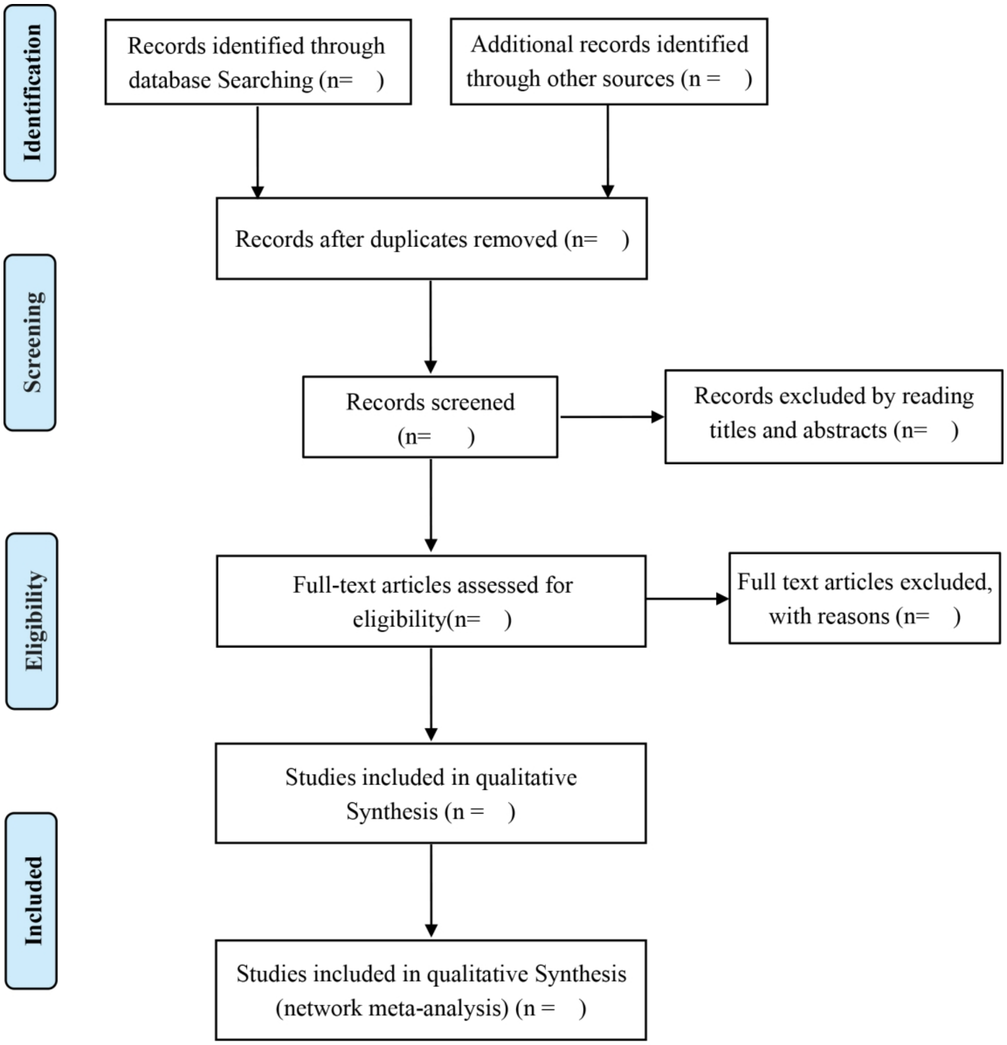

PRISMA flow chart of study selection process.

161x169mm (300 x 300 DPI)

PRISMA-P (Preferred Reporting Items for Systematic review and Meta-Analysis Protocols) 2015 checklist: recommended items to address in a systematic review protocol*

Section and topic	Item No	Checklist item	Reported on Page #
**ADMINISTRATIVE INFORMATION**			
Title:			
Identification	1a	Identify the report as a protocol of a systematic review	1
Update	1b	If the protocol is for an update of a previous systematic review, identify as such	
Registration	2	If registered, provide the name of the registry (such as PROSPERO) and registration number	2
Authors:			
Contact	3a	Provide name, institutional affiliation, e-mail address of all protocol authors; provide physical mailing address of corresponding author	1
Contributions	3b	Describe contributions of protocol authors and identify the guarantor of the review	9
Amendments	4	If the protocol represents an amendment of a previously completed or published protocol, identify as such and list changes; otherwise, state plan for documenting important protocol amendments	
Support:			
Sources	5a	Indicate sources of financial or other support for the review	9
Sponsor	5b	Provide name for the review funder and/or sponsor	
Role of sponsor or funder	5c	Describe roles of funder(s), sponsor(s), and/or institution(s), if any, in developing the protocol	
**INTRODUCTION**			
Rationale	6	Describe the rationale for the review in the context of what is already known	
Objectives	7	Provide an explicit statement of the question(s) the review will address with reference to participants, interventions, comparators, and outcomes (PICO)	4-5
**METHODS**			
Eligibility criteria	8	Specify the study characteristics (such as PICO, study design, setting, time frame) and report characteristics (such as years considered, language, publication status) to be used as criteria for eligibility for the review	4-5
Information sources	9	Describe all intended information sources (such as electronic databases, contact with study authors, trial registers or other grey literature sources) with planned dates of coverage	4
Search strategy	10	Present draft of search strategy to be used for at least one electronic database, including planned limits, such that it could be repeated	4

For peer review only

Study records:			
Data management	11a	Describe the mechanism(s) that will be used to manage records and data throughout the review	6
Selection process	11b	State the process that will be used for selecting studies (such as two independent reviewers) through each phase of the review (that is, screening, eligibility and inclusion in meta-analysis)	6
Data collection process	11c	Describe planned method of extracting data from reports (such as piloting forms, done independently, in duplicate), any processes for obtaining and confirming data from investigators	6
Data items	12	List and define all variables for which data will be sought (such as PICO items, funding sources), any pre-planned data assumptions and simplifications	6
Outcomes and prioritization	13	List and define all outcomes for which data will be sought, including prioritization of main and additional outcomes, with rationale	5
Risk of bias in individual studies	14	Describe anticipated methods for assessing risk of bias of individual studies, including whether this will be done at the outcome or study level, or both; state how this information will be used in data synthesis	6
Data synthesis	15a	Describe criteria under which study data will be quantitatively synthesised	7-8
	15b	If data are appropriate for quantitative synthesis, describe planned summary measures, methods of handling data and methods of combining data from studies, including any planned exploration of consistency (such as I^2, Kendall's τ)	7
	15c	Describe any proposed additional analyses (such as sensitivity or subgroup analyses, meta-regression)	8
	15d	If quantitative synthesis is not appropriate, describe the type of summary planned	
Meta-bias(es)	16	Specify any planned assessment of meta-bias(es) (such as publication bias across studies, selective reporting within studies)	8
Confidence in cumulative evidence	17	Describe how the strength of the body of evidence will be assessed (such as GRADE)	8

For peer review only

Dear Editor:

We would like to submit the manuscript entitled “The comparative efficacy of different acupuncture as adjuvant therapy on carotid atherosclerosis: A protocol for systematic review and network meta-analysis”, which we wish to be considered for publication in “BMJ Open”. In this work, we evaluated the effects of different types of acupuncture treatments on CAS, and a ranking of the therapeutic classes will also be presented, aiming to provide evidence-based medicine for its extensive clinical application.

No conflict of interest exists in the submission of this manuscript, and the manuscript is approved by all authors for publication. I would like to declare on behalf of my co-authors that the work described was original research that has not been published previously, and not under consideration for publication elsewhere, in whole or in part. All the authors listed have approved the manuscript that is enclosed.

We deeply appreciate your consideration of our manuscript, and we look forward to receiving comments from the reviewers. If you have any queries, please don’t hesitate to contact me at the address below.

Thank you and best regards.

Yours sincerely,

Name: Xianming Wu or Qian Mo

Phone: +86-15502312023 or +86-18286180926

E-mail: 564339014@qq.com or duoduo425@126.com

For peer review only

BMJ Open

The comparative efficacy of different acupuncture as adjuvant therapy on carotid atherosclerosis: A protocol for systematic review and network meta-analysis

Journal:	*BMJ Open*
Manuscript ID	bmjopen-2021-049778.R1
Article Type:	Protocol
Date Submitted by the Author:	20-Nov-2021
Complete List of Authors:	Wu, xianming; Hunan University of Chinese Medicine<br>Mo, Qian; Guizhou University of Traditional Chinese Medicine<br>Yang, Zhihong; Guizhou University of Traditional Chinese Medicine<br>Huang, Xiaolou; Guizhou University of Traditional Chinese Medicine<br>Liu, Jiao; Guizhou University of Traditional Chinese Medicine<br>Xu, Shuangmei; Guizhou University of Traditional Chinese Medicine<br>Zhang, Ning; Guizhou University of Traditional Chinese Medicine<br>Yang, Xiaofang; Hunan University of Chinese Medicine; Guizhou University Of Traditional Chinese Medicine
**Primary Subject Heading**:	Cardiovascular medicine
Secondary Subject Heading:	Public health
Keywords:	COMPLEMENTARY MEDICINE, CARDIOLOGY, VASCULAR MEDICINE

SCHOLARONE™
Manuscripts

The comparative efficacy of different acupuncture as adjuvant therapy on carotid atherosclerosis: A protocol for systematic review and network meta-analysis

Xianming Wu,[1] Qian Mo,[2] Zhihong Yang,[2] Xiaolou Huang,[2] Jiao Liu,[2] Shuangmei Xu,[2] Ning Zhang,[2*] Xiaofang Yang,[1, 2 *]

[1] Hunan University of Chinese Medicine, Changsha, China

[2] Guizhou University of Traditional Chinese Medicine, Guiyang, China

XW and QM contributed equally to this paper.

Correspondence to:

Ning Zhang and Xiaofang Yang; zhangning_nico@163.com; Yangxiaofang210@163.com;

Author's address: Dongqing South Road, Huaxi University Town, Gui'an New District, Guizhou Province, China.

For peer review only

Abstract

Introduction Carotid atherosclerosis (CAS) is a disease of the aorta caused by lipid metabolism disorders and local inflammation. Acupuncture combined with traditional western medicine (such as aspirin or atorvastatin) for the treatment of CAS has been widely applied in clinical practice, but there is still a lack of supporting evidence for the efficacy and safety of CAS. Therefore, this systematic review and network meta-analysis (NMA) will summarize the effects of different types of acupuncture treatments on CAS, and a ranking of the therapeutic classes will also be presented, aiming to provide evidence-based medicine for its extensive clinical application.

Methods and analysis Systematic and NMA searches will be conducted in seven electronic databases, PubMed, EMBASE, Medline, Cochrane Library, Chinese National Knowledge Infrastructure, Wan fang Database, and Chongqing VIP databases (CQVIP). The search time is from their inception to December 2020, regardless of language and publication type. Randomized controlled trials (RCTs) and controlled clinical trials (CCTs) that include patients with CAS receiving acupuncture therapy compared with a control group will be considered eligible. The primary outcomes include the carotid intima-media thickness (cIMT) and vessel plaque quantification (VPQ), the secondary outcomes include the carotid plaque Crouse score grey-scale median (GSM), lipid levels, the incidence of cardiovascular events, safety, and adverse events. The selection of studies, data extraction, quality assessment, and risk of bias assessment will be conducted by two independent reviewers. The NMA will be analyzed with Stata V.15.0, RevMan V.5.3 software, and WinBUGS 1.4.3.

Ethics and dissemination Ethical approval will not be required for this study as it will be based on de-identified, aggregate published data. We will publish the findings in a peer-reviewed journal.

Patient and Public Involvement: No patient involved.

PROSPERO registration number CRD42020207260.

Keywords: Carotid atherosclerosis; Acupuncture; adjuvant therapy; systematic review; network meta-analysis

For peer review only - http://bmjopen.bmj.com/site/about/guidelines.xhtml

Strengths and limitations of this study

1. This study will be the first comprehensive analysis of the efficacy of acupuncture as an adjuvant therapy in the treatment of atherosclerosis. And a ranking of the therapeutic classes will also be presented.
2. The data report will follow the Preferred Reporting Items of the System review and the Meta-analysis guidelines.
3. This study only includes English and Chinese trials, which may lead to the potential risk of ignoring some studies.
4. The heterogeneity of different studies may affect the final results of this study.

For peer review only

For peer review only - http://bmjopen.bmj.com/site/about/guidelines.xhtml

INTRODUCTION

Atherosclerosis (AS) is a series of pathological changes in the vascular intima through lipid infiltration, platelet activation, thrombosis, intimal injury, inflammatory response, oxidative stress, and activation of vascular smooth muscle cells (VSMC). It is manifested by cell metabolism disorder, product accumulation, which causes the vessel wall to harden and thicken, and lose its elasticity, the inner diameter of the lumen becomes smaller, blood flow is blocked, and finally, the diseased blood vessel blood supply organs and even life are endangered.[1-3] The World Health Organization (WHO) reports that non-communicable diseases cause 41 million deaths each year, equivalent to 71% of the total global deaths. Among them, cardiovascular diseases cause 17.9 million deaths each year.[4] Globally, the number of people dying from cardiovascular disease is still increasing. WHO plans to reduce premature mortality from non-communicable diseases by one-third by 2030 through treatment and prevention.[4] Among them, carotid artery atherosclerosis (CAS) has common risk factors and pathological basis with atherosclerosis of the coronary arteries of the heart, cerebral blood vessels, and renal arteries.[5-7] A study has found that 93% of cerebral infarctions are accompanied by CAS, and 18%-25% of ischemic strokes can be attributed to thromboembolism caused by CAS[8]. cIMT is related to the risk of cardiovascular events in the general population, and cIMT measurement is essential for assessing the risk and incidence of cardiovascular disease.[9] Of course, VPQ is a non-invasive evaluation method for observing the morphological characteristics of plaques, and it is also regarded as the main observation in clinical practice.[10]

The current conventional Western medicine treatments for carotid atherosclerosis mainly consist of lifestyle modification, drug therapy, etc. Lifestyle modification includes smoking cessation, control of energy intake, etc. The drugs therapy includes hypolipidemic, antiplatelet, etc. (such as aspirin or atorvastatin).[11] Statins can treat AS by regulating blood lipids, improving vascular endothelial function, inhibiting thrombosis, anti-inflammatory, anti-oxidation, and stabilizing plaque.[12] Acupuncture is part of the characteristic treatment of traditional Chinese medicine. Its main feature is to stimulate the meridians and acupoints and regulate the qi and blood of the viscera to achieve the purpose of disease prevention and treatment, it has the effects of anti-inflammation, immunity activation and nervous system

For peer review only - http://bmjopen.bmj.com/site/about/guidelines.xhtml

modulation.[13,14] At present, acupuncture is widely used as an adjuvant treatment of AS as a characteristic treatment method of Chinese medicine.[15,16] One of the mechanisms of electroacupuncture treatment of atherosclerosis may be by reducing the expression of CD36 protein and mRNA in AS rabbit macrophages.[17]

Although there have been RCTs and CCTs to study the efficacy of acupuncture intervention on AS, due to sample size and insufficient randomization methods in clinical trial studies, and the different objectives and outcome evaluation indicators used, the results of the research are not consistent. What type of acupuncture method is better to prevent, stabilize and reverse CAS is still inconclusive. Therefore, this study uses a network meta-analysis to summarize the effects of different types of acupuncture treatments on CAS and aims to provide evidence-based medicine for the extensive clinical use of acupuncture as an adjuvant therapy to treat AS.

OBJECTIVE

The objectives of our study are:

1. To evaluate the efficacy and safety of acupuncture as adjuvant therapy in the treatment of AS.

2. To present the ranking of therapeutic classes.

METHODS

Study registration

This NMA will be performed according to the guidelines of preferred reporting items for systematic reviews and meta-analyses (PRISMA-NMA) statement.[18] The protocol has been registered on the PROSPERO website (https://www.crd.york.ac.uk/prospero/) and the PROSPERO registration number is CRD42020207260.

Patient and public involvement

No patient or public will be involved in the study directly.

Data sources and search strategy

For peer review only

For peer review only - http://bmjopen.bmj.com/site/about/guidelines.xhtml

Seven electronic databases including PubMed, EMBASE, Medline, Cochrane Library, CNKI, Wan fang Database, and CQVIP will be searched from inception to December 2020. The search strategy will be adjusted according to the characteristics of the database. The detailed search strategy of PubMed will be shown in Table 1, while other databases are shown in online supplemental appendix 1.

Inclusion and exclusion criteria

Types of studies

All RCTs and CCTs will be included and the full text is available, conference proceedings, dissertations might be included if feasible, irrespective of language and publication. Retrospective studies, case reports, reviews, or studies on the mechanism of action will be excluded.

Participants

Studies that enrolled patients were diagnosed with CAS, without serious liver and kidney dysfunction and tumor patients. while gender, age, ethnicity, and nationality will not be limited. The baseline data of the observation group and the control group are comparable. We will follow the relevant standards of the guidelines for atherosclerotic vascular ultrasound examination[11].

Types of interventions

Both the observation group and the control group were treated with conventional western medicine, including aspirin or atorvastatin, etc. The intervention of the observation group was to add acupuncture to the conventional Western medicine treatment. Acupuncture therapies included acupuncture, electro-acupuncture, auricular acupuncture, warm acupuncture, etc., without restrictions of acupuncture operation methods, acupoint selection, duration of treatment, and follow-up period.

Types of comparison

For peer review only - http://bmjopen.bmj.com/site/about/guidelines.xhtml

The control group will include treatments of placebo or the same western medicine treatment in the observation group, such as aspirin or atorvastatin.

Outcomes

The primary outcome indicator will be the changes of cIMT and VPQ before and after treatment.

The secondary outcomes include the changes of carotid plaque Crouse score, GSM, lipid levels. Safety outcomes include the type and frequency of occurrence of adverse reactions, etc. such as subcutaneous hematoma, fatigue, palpitations, etc.

Selection of studies.

According to the inclusion and exclusion criteria, two independent reviewers (XMW and QM) will remove duplicates and unqualified articles after reading the titles and abstract. For articles that meet the inclusion criteria, the full texts will be evaluated for eligibility. If there is any disagreement between the two reviewers, the third reviewer (XFY) will make a final decision after discussion. If the data or information are incomplete, the authors will be contacted. The PRISMA flow diagram (Figure 1) will be used to show the research screening process.

Data extraction and management

All reviewers discussed and developed data extraction forms based on the Cochrane Handbook, and two independent reviewers (XMW and QM) will extract data from the included studies. The extracted data includes general information (author, publication year, journal, etc.), participant, sample size, intervention methods, duration, outcomes, follow-up time (Table 2). If the data is incomplete, the corresponding author will be contacted. Multi-arm trials will be assigned into two-arm to ensure that the results can be combined.[19]

Risk of bias assessment

Two reviewers (XMW and QM) will use the "Risk of bias" tool from the Cochrane Handbook (V.5.1.0) to assess the quality of the included studies.[18,20] The contents of the evaluation include random sequence generation, allocation concealment, and implementation of

For peer review only - http://bmjopen.bmj.com/site/about/guidelines.xhtml
For peer review only

blinding, incomplete outcome data, selective reports, and other issues. Any inconsistency will be decided through discussion with the third reviewer (XFY). The risk of bias will be classified as "high risk of bias", "low risk of bias" or "unclear risk of bias".

STATISTICAL ANALYSIS

Standard pairwise meta-analysis

Standard pairwise meta-analysis was performed using Stata V.15.0 software. The odds ratio (OR) was used as the effect size for count data, the mean difference was used for measurement data, and 95% confidence interval (CI) was used for interval estimation. According to the "Cochrane Handbook", the Mantel-Haenszel χ^2 test and Higgins I^2 test will analyze the heterogeneity. *P*-value <.10 or I^2 >50% indicates that the heterogeneity is statistically significant, and the source of the heterogeneity is analyzed. And according to the possible heterogeneity factors, conduct subgroup analysis, and then sensitivity analysis was carried out to ascertain the reliability of the data, find abnormal studies that lead to significant heterogeneity, and analyze the reasons. Heterogeneity also depends on the size of the impact, the direction of the results, and the strength of the evidence.

Network meta-analysis and network geometry

WinBUGS 1.4.3 will be used for network statistical analysis,[21] with a random effect model containing direct comparison and indirect comparison, and the calculation will be performed by the Bayesian Markov chain Monte Carlo methods. The number of iterations is 50000, the 10000 are used for annealing to eliminate the influence of the initial value, and sampling starts after 10001. Calculate the surface under cumulative ranking area (SUCRA) to predict the curative effect of each treatment measure and rank it. The result is expressed as a percentage, the larger the value, the better the curative effect of the intervention.[22]

Stata 15.0 draws a network geometry, the results of different types of acupuncture effects will be demonstrated: The line between the two circles indicates that there is a direct correlation between the two interventions, and the absence of a line means that there is no direct correlation, and only indirect comparisons can be made. The thickness of the line represents the number of direct comparisons studies, the area of the circle represents the

For peer review only - http://bmjopen.bmj.com/site/about/guidelines.xhtml

number of intervention studies evaluated.[23,24] The comparisons adjusted funnel plot was used to compare sample effects between studies and evaluate the publication bias of the included studies.

Assessment of inconsistency

The basic principle of NMA is that the included studies are consistent, and the results of indirect comparison and direct comparison should be similar. We will use loop-specific methods to detect loops of evidence that may have important inconsistencies.[25] A node split model is used to compare the consistency of direct comparison and indirect comparison,[26] P>0.05 means that the consistency between direct and indirect comparison is better, consistency model is used for analysis. Otherwise, the inconsistency model is used. For those that have not generated node split, the consistency model is used for analysis.

Subgroup and sensitivity analyses

To assess the impact of covariates in heterogeneity, such as gender, disease severity, we will explore subgroup analysis.

Subgroup analysis still has obvious heterogeneity, and sensitivity analysis will be performed. Eliminate low-quality studies one by one, and then merge the data. The meta-analysis will be repeated and compare the results of the two meta-analyses to identify the impact of each study on the overall results.

Assessment of publication biases

If more than 10 trials are included, a Deek's funnel plot to evaluate publication bias. If the angle between the regression line and the X-axis is closer to 90°, it means that the less possibility of publication bias.[27]

Ethics and dissemination

Since the study will be based on de-identified, aggregate published data, ethical approval is not required. The final report of this review will be disseminated through peer-reviewed publications or conference reports.

For peer review only - http://bmjopen.bmj.com/site/about/guidelines.xhtml

DISCUSSION

CAS is a common multiple diseases that endangers human health. The common pathological basis of cardiovascular and cerebrovascular diseases has become the main cause of death in the world. Clinically, the treatment of AS is roughly divided into drugs and non-drug treatments. Among them, drug treatments include statins, which have anti-platelet, anti-inflammatory, lipid-regulating, and inhibit angiogenesis effects in plaques.[28,29] Although great progress has been made in the treatment of AS, the complex and diverse causes and mechanisms of AS (closely related to metabolic, environmental, genetic, and other factors) make the existing clinical intervention methods. The treatment of AS is still insufficient, and diseases caused by AS still bring huge health and economic burden to society.[30] In recent years, acupuncture has been widely used as an adjuvant treatment of AS. However, there is no research to rank acupuncture interventions and evaluate the optimal acupuncture methods. Therefore, this network meta-analysis will conduct a detailed summary and analysis of acupuncture as adjuvant therapy for CAS to determine the optimal acupuncture therapy.

The comparison between therapies in the network meta-analysis is based on direct comparisons of interventions within RCTs and indirect comparisons across trials based on a common comparator, such as placebo or some standard treatment. The interrelationship between multiple interventions can be analyzed, and deal with factors that may have an impact due to the number of included articles, heterogeneity, and inconsistency.[31,32]

Nevertheless, this network meta-analysis still has limitations. Some low-quality trials may affect the final results of the NMA. In addition, the heterogeneity of different studies may affect the final results of this study. Finally, this study only includes English and Chinese trials, which may lead to the potential risk of ignoring some studies.

Despite the limitations, the latest data should be systematically reviewed. It is concluded that different types of acupuncture can assist patients with CAS to better improve carotid IMT, and can provide evidence-based medicine for clinical evidence.

Contributors XW and QM conceived of the protocol and drafted the manuscript. XW and JL registered the protocol review in the PROSPERO, ZY and XH developed the search strategy, NZ and

For peer review only - http://bmjopen.bmj.com/site/about/guidelines.xhtml

SX revised the manuscript for methodological and intellectual content. XY contributed to and approved the final manuscript of the protocol review.

Funding This study is supported by National Natural Science Foundation of China (grant number: 82160941, 82160937).

Competing interests None declared.

Patient and public involvement Patients and/or the public were not involved in the design, or conduct, or reporting, or dissemination plans of this research.

Patient consent for publication Not required.

Provenance and peer review Not commissioned; externally peer reviewed.

Open access This is an open access article distributed in accordance with the Creative Commons Attribution Non Commercial (CC BY- NC 4.0) license, which permits others to distribute, remix, adapt, build upon this work non- commercially, and license their derivative works on different terms, provided the original work is properly cited, appropriate credit is given, any changes made indicated, and the use is non- commercial. See: http:// creativecommons. org/ licenses/ by- nc/ 4. 0/.References

ORCID iD Xianming Wu https://orcid.org/0000-0002-4825-0944

REFERENCES

1. Xu S, Kamato D, Little PJ, Nakagawa S, Pelisek J, Jin ZG. Targeting epigenetics and non-coding RNAs in atherosclerosis: from mechanisms to therapeutics. *Pharmacology & therapeutics.* 2019;196:15-43.
2. Zhu Y, Xian X, Wang Z, et al. Research Progress on the Relationship between Atherosclerosis and Inflammation. *Biomolecules.* 2018;8(3).
3. Tall AR, Yvan-Charvet L. Cholesterol, inflammation and innate immunity. *Nature reviews Immunology.* 2015;15(2):104-116.
4. Origination WH. Noncommunicable diseases. 2021, April 13 Retrieved from https://www.who.int/zh/news-room/fact-sheets/detail/noncommunicable-diseases.
5. Zhao D, Liu J, Wang M, Zhang X, Zhou M. Epidemiology of cardiovascular disease in China: current features and implications. *Nature reviews Cardiology.* 2019;16(4):203-212.
6. Kobiyama K, Ley K. Atherosclerosis. *Circulation research.* 2018;123(10):1118-1120.
7. Libby P, Buring JE, Badimon L, et al. Atherosclerosis. *Nature reviews Disease primers.*

For peer review only - http://bmjopen.bmj.com/site/about/guidelines.xhtml

2019;5(1):56.
8. Ooi YC, Gonzalez NR. Management of extracranial carotid artery disease. *Cardiology clinics.* 2015;33(1):1-35.
9. Cao JJ, Arnold AM, Manolio TA, et al. Association of carotid artery intima-media thickness, plaques, and C-reactive protein with future cardiovascular disease and all-cause mortality: the Cardiovascular Health Study. *Circulation.* 2007;116(1):32-38.
10. Zhou J, Zhao L, Meng L, et al. Acupuncture treatment for carotid atherosclerotic plaques: study protocol for a pilot randomized, single blinded, controlled clinical trial. *Trials.* 2020;21(1):768.
11. Kinoshita M, Yokote K, Arai H, et al. Japan Atherosclerosis Society (JAS) Guidelines for Prevention of Atherosclerotic Cardiovascular Diseases 2017. *Journal of atherosclerosis and thrombosis.* 2018;25(9):846-984.
12. Liu M, Liu G, Wang M. Comparison and analysis of the therapeutic effect of different statins in the treatment of atherosclerosis. *Pakistan journal of pharmaceutical sciences.* 2018;31.
13. Zhang YP LY, Xu Y, et al. Preliminary Analysis on Anti-inflammatory Mechanism of Acupuncture. *Journal of Clinical Acupuncture and Moxibustion* 2018;34(01):77-80.
14. Han Z, Zhang Y, Wang P, Tang Q, Zhang K. Is acupuncture effective in the treatment of COVID-19 related symptoms? Based on bioinformatics/network topology strategy. *Briefings in bioinformatics.* 2021;22(5).
15. Shen Y, Cheng ZD, Chen YG, et al. Electroacupuncture Inhibits Atherosclerosis through Regulating Intestinal Flora and Host Metabolites in Rabbit. *Evidence-based complementary and alternative medicine : eCAM.* 2020;2020:5790275.
16. HT L, LC M, Y Y, et al. Clinical Observation on Acupuncture Treatment of Carotid Atherosclerosis of Phlegm-Damp Constitution. *Guangdong Medical Journal.* 2018;39(13):2070-2073.
17. Cheng Z, Ning Y, Wang R. [Effects of electroacupuncture on expression of CD36 in peritoneal macrophages of rabbits with atherosclerosis]. *Zhongguo zhen jiu = Chinese acupuncture & moxibustion.* 2018;38(2):179-184.
18. Julian PT Higgins SG. Cochrane Handbook for Systematic Reviews of Interventions Version 5.1.0 2011.
19. Zhang C, Yan JZ, Sun F, Liu Q, Zeng XT. Dif erentiation and Handling of Homogeneity in Network Meta-analysis. *Chinese Journal of Evidence-Based Medicine.* 2014;14(7):884-888.
20. Guyatt G, Busse J. Tools to assess risk of bias in randomized controlled trials. In:2015.
21. Dave V, Yadav RB, Yadav S, Sharma S, Sahu RK, Ajayi AF. A Critique of Computer Simulation Software's Used in Pharmacokinetics and Pharmacodynamics Analysis. *Current clinical pharmacology.* 2018;13(4):216-235.
22. Cope S, Jansen JP. Quantitative summaries of treatment effect estimates obtained with network meta-analysis of survival curves to inform decision-making. *BMC medical research methodology.* 2013;13:147.
23. van Valkenhoef G, Lu G, de Brock B, Hillege H, Ades AE, Welton NJ. Automating network meta-analysis. *Research synthesis methods.* 2012;3(4):285-299.
24. Nyaga VN, Aerts M, Arbyn M. ANOVA model for network meta-analysis of diagnostic test accuracy data. *Statistical methods in medical research.* 2018;27(6):1766-1784.
25. Bucher HC, Guyatt GH, Griffith LE, Walter SD. The results of direct and indirect treatment

For peer review only - http://bmjopen.bmj.com/site/about/guidelines.xhtml

comparisons in meta-analysis of randomized controlled trials. *Journal of clinical epidemiology.* 1997;50(6):683-691.

26. Dias S, Welton NJ, Caldwell DM, Ades AE. Checking consistency in mixed treatment comparison meta-analysis. *Statistics in medicine.* 2010;29(7-8):932-944.
27. Deeks JJ, Macaskill P, Irwig L. The performance of tests of publication bias and other sample size effects in systematic reviews of diagnostic test accuracy was assessed. *Journal of clinical epidemiology.* 2005;58(9):882-893.
28. Kirichenko TV, Sobenin IA, Nikolic D, Rizzo M, Orekhov AN. Anti-cytokine therapy for prevention of atherosclerosis. *Phytomedicine : international journal of phytotherapy and phytopharmacology.* 2016;23(11):1198-1210.
29. Khosravi M, Poursaleh A, Ghasempour G, Farhad S, Najafi M. The effects of oxidative stress on the development of atherosclerosis. *Biological chemistry.* 2019;400(6):711-732.
30. Bubnova MG, Kukharchuk VV. [Recommendations of the European Society of Cardiology and the European Atherosclerosis Society on Cardiovascular Disease Prevention and Management of Dyslipidemias. for the Diagnosis of Atherosclerosis and Dyslipidemia Treatment (2016): Basic S.G.]. *Kardiologiia.* 2017;57(3):85-89.
31. Li T, Puhan MA, Vedula SS, Singh S, Dickersin K. Network meta-analysis-highly attractive but more methodological research is needed. *BMC medicine.* 2011;9:79.
32. Mills EJ, Thorlund K, Ioannidis JP. Demystifying trial networks and network meta-analysis. *BMJ (Clinical research ed).* 2013;346:f2914.

For peer review only

Table

Table 1 Search strategy for PubMed database	
Number	Search items
#1	Atherosclerosis [MeSH]
#2	Atheroscleroses [Title/Abstract]
#3	Atherogenesis [Title/Abstract]
#4	Carotid atherosclerosis [Title/Abstract]
#5	#1 or #2 or #3 or #4
#6	Acupuncture [MeSH]
#7	Acupuncture Therapy [Title/Abstract]
#8	Acupuncture, Ear [Title/Abstract]
#9	Acupuncture Points [Title/Abstract]
#10	Electroacupuncture [Title/Abstract]
#11	Auricular point [Title/Abstract]
#12	#6 or #7 or #8 or #9 or #10 or #11
#13	Statins [Title/Abstract]
#14	atorvastatin [Title/Abstract]
#15	aspirin [Title/Abstract]
#16	#13 or #14 or #15
#17	randomized controlled trial [Publication Type]
#18	randomized [Title/Abstract]
#19	randomly [Title/Abstract]
#20	controlled clinical trial [Title/Abstract]
#21	clinical trial [Title/Abstract]
#22	trial [Title/Abstract]
#23	#17 or #18 or #19 or #20 or #21 or #22
#24	#5 and #12 and #16 and #23

MeSH: Medical Subject Headings

Table 2 Characteristics of included studies

Reference	Country	Study design	Population		Intervention			Outcomes	Adverse event	Flow-up
			Sample age	Sample size	I	C	Duration			

I：Intervention; C: Control

For peer review only - http://bmjopen.bmj.com/site/about/guidelines.xhtml

Figure legend

Fig.1 PRISMA flow chart of study selection process.

For peer review only - http://bmjopen.bmj.com/site/about/guidelines.xhtml

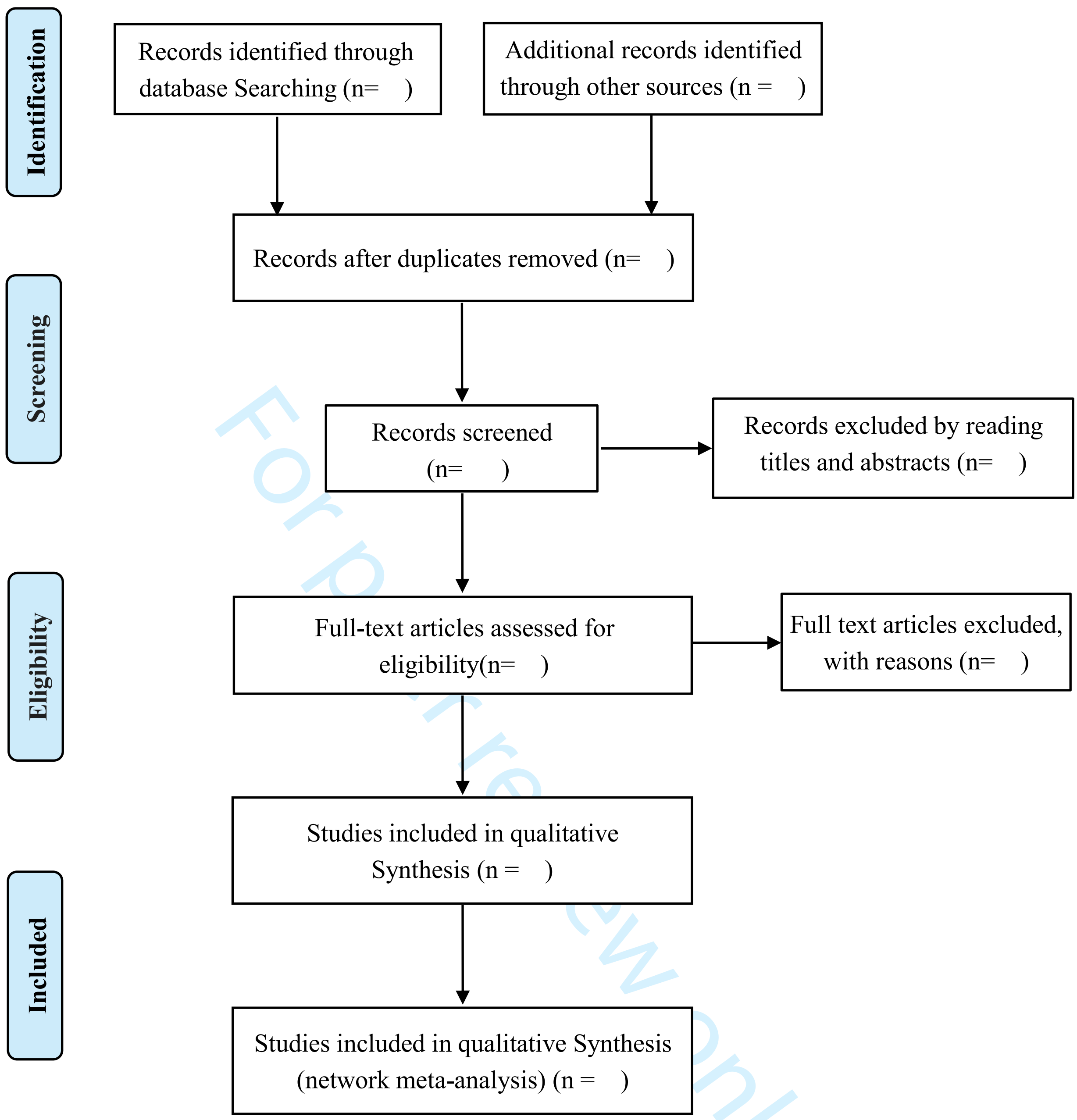

Figure 1. PRISMA flow chart of study selection process.

Search strategy for EMBASE	
Number	Search items
#1	"Atherosclerosis"/exp
#2	Atheroscleroses: ab,ti
#3	Atherogenesis: ab,ti
#4	"Carotid atherosclerosis": ab,ti
#5	#1 or #2 or #3 or #4
#6	"Acupuncture"/exp
#7	"Acupuncture Therapy": ab,ti
#8	"Acupuncture, Ear": ab,ti
#9	"Acupuncture Points": ab,ti
#10	Electroacupuncture: ab,ti
#11	"Auricular point": ab,ti
#12	#6 or #7 or #8 or #9 or #10 or #11
#13	"Statins"/exp
#14	atorvastatin: ab,ti
#15	aspirin: ab,ti
#16	#13 or #14 or #15
#17	"randomized controlled trial"/exp
#18	"randomized controlled trial": ab,ti
#19	randomized: ab,ti
#20	randomly: ab,ti
#21	"controlled clinical trial": ab,ti
#22	"clinical trial": ab,ti
#23	trial: ab,ti
#24	#17 or #18 or #19 or #20 or #21 or # 22# 23
#25	#5 and #12 and #16 and # 24

For peer review only

Search strategy for Medline database						
Atherosclerosis		Acupuncture		Statins		'Randomized Controlled Trial'
OR		OR		OR		OR
Atheroscleroses		'Acupuncture Therapy'		Atorvastatin		randomized
OR		OR		OR		OR
Atherogenesis	AND	'Acupuncture, Ear'	AND	Aspirin	AND	randomly
OR		OR				OR
'Carotid		'Acupuncture Points'				'Controlled Clinical Trial'
atherosclerosis'		OR				OR
		'Electroacupuncture'				'clinical trial'
		OR				OR
		'Auricular point'				trial

For peer review only

Search strategy for Cochrane Library	
Number	Search items
#1	MeSH descriptor: [Atherosclerosis] explode all trees
#2	Atheroscleroses: ti,ab,kw
#3	Atherogenesis: ti,ab,kw
#4	“Carotid atherosclerosis”: ti,ab,kw
#5	#1 or #2 or #3 or #4
#6	MeSH descriptor: [Acupuncture] explode all trees
#7	“Acupuncture Therapy”: ti,ab,kw
#8	“Acupuncture, Ear”: ti,ab,kw
#9	“Acupuncture Points”: ti,ab,kw
#10	Electroacupuncture: ti,ab,kw
#11	“Auricular point”: ti,ab,kw
#12	#6 or #7 or #8 or #9 or #10 or #11
#13	MeSH descriptor: [Statins] explode all trees
#14	atorvastatin: ti,ab,kw
#15	aspirin: ti,ab,kw
#16	#13 or #14 or #15
#17	MeSH descriptor: [Randomized Controlled Trial] explode all trees
#18	“Randomized Controlled Trial”: ti,ab,kw
#19	randomized: ti,ab,kw
#20	randomly: ti,ab,kw
#21	“controlled clinical trial”: ti,ab,kw
#22	“clinical trial”: ti,ab,kw
#23	trial: ti,ab,kw
#24	#17 or #18 or #19 or #20 or #21 or # 22# 23
#25	#5 and #12 and #16 and # 24

For peer review only

Search strategy for CNKI
（主题或摘要：动脉粥样硬化+动脉硬化+斑块+颈动脉粥样硬化+颈动脉硬化+颈动脉）AND（主题或摘要：针刺+针灸+电针+温针灸+耳针+耳穴+穴位）AND（主题或摘要：他汀类药物+阿司匹林）AND（主题或摘要：随机对照研究+随机+对照研究+对照+试验+临床研究）

Search strategy for WF
（主题：动脉粥样硬化+动脉硬化+斑块+颈动脉粥样硬化+颈动脉硬化+颈动脉）AND（主题：针刺+针灸+电针+温针灸+耳针+耳穴+穴位）AND（主题：他汀类药物+阿司匹林）AND（主题：随机对照研究+随机+对照研究+对照+试验+临床研究）

Search strategy for CQVIP
（题名或关键词：动脉粥样硬化+动脉硬化+斑块+颈动脉粥样硬化+颈动脉硬化+颈动脉）AND（题名或关键词：针刺+针灸+电针+温针灸+耳针+耳穴+穴位）AND（题名或关键词：他汀类药物+阿司匹林）AND（题名或关键词：随机对照研究+随机+对照研究+对照+试验+临床研究）

For peer review only

PRISMA-P (Preferred Reporting Items for Systematic review and Meta-Analysis Protocols) 2015 checklist: recommended items to address in a systematic review protocol*

Section and topic	Item No	Checklist item	Reported on Page #
**ADMINISTRATIVE INFORMATION**			
Title:			
Identification	1a	Identify the report as a protocol of a systematic review	1
Update	1b	If the protocol is for an update of a previous systematic review, identify as such	
Registration	2	If registered, provide the name of the registry (such as PROSPERO) and registration number	2
Authors:			
Contact	3a	Provide name, institutional affiliation, e-mail address of all protocol authors; provide physical mailing address of corresponding author	1
Contributions	3b	Describe contributions of protocol authors and identify the guarantor of the review	10-11
Amendments	4	If the protocol represents an amendment of a previously completed or published protocol, identify as such and list changes; otherwise, state plan for documenting important protocol amendments	
Support:			
Sources	5a	Indicate sources of financial or other support for the review	11
Sponsor	5b	Provide name for the review funder and/or sponsor	
Role of sponsor or funder	5c	Describe roles of funder(s), sponsor(s), and/or institution(s), if any, in developing the protocol	
**INTRODUCTION**			
Rationale	6	Describe the rationale for the review in the context of what is already known	
Objectives	7	Provide an explicit statement of the question(s) the review will address with reference to participants, interventions, comparators, and outcomes (PICO)	6-7
**METHODS**			
Eligibility criteria	8	Specify the study characteristics (such as PICO, study design, setting, time frame) and report characteristics (such as years considered, language, publication status) to be used as criteria for eligibility for the review	6-7
Information sources	9	Describe all intended information sources (such as electronic databases, contact with study authors, trial registers or other grey literature sources) with planned dates of coverage	5-6
Search strategy	10	Present draft of search strategy to be used for at least one electronic database, including planned limits, such that it could be repeated	5-6

For peer review only

Study records:			
Data management	11a	Describe the mechanism(s) that will be used to manage records and data throughout the review	7
Selection process	11b	State the process that will be used for selecting studies (such as two independent reviewers) through each phase of the review (that is, screening, eligibility and inclusion in meta-analysis)	7
Data collection process	11c	Describe planned method of extracting data from reports (such as piloting forms, done independently, in duplicate), any processes for obtaining and confirming data from investigators	7
Data items	12	List and define all variables for which data will be sought (such as PICO items, funding sources), any pre-planned data assumptions and simplifications	7
Outcomes and prioritization	13	List and define all outcomes for which data will be sought, including prioritization of main and additional outcomes, with rationale	7
Risk of bias in individual studies	14	Describe anticipated methods for assessing risk of bias of individual studies, including whether this will be done at the outcome or study level, or both; state how this information will be used in data synthesis	7-8
Data synthesis	15a	Describe criteria under which study data will be quantitatively synthesised	8-9
	15b	If data are appropriate for quantitative synthesis, describe planned summary measures, methods of handling data and methods of combining data from studies, including any planned exploration of consistency (such as I^2, Kendall's τ)	8
	15c	Describe any proposed additional analyses (such as sensitivity or subgroup analyses, meta-regression)	9
	15d	If quantitative synthesis is not appropriate, describe the type of summary planned	
Meta-bias(es)	16	Specify any planned assessment of meta-bias(es) (such as publication bias across studies, selective reporting within studies)	9
Confidence in cumulative evidence	17	Describe how the strength of the body of evidence will be assessed (such as GRADE)	7-8

For peer review only
